# An exploration of alternative visualisations of the basic helix-loop-helix protein interaction network

**DOI:** 10.1186/1471-2105-8-289

**Published:** 2007-08-06

**Authors:** Brian J Holden, John W Pinney, Simon C Lovell, Grigoris D Amoutzias, David L Robertson

**Affiliations:** 1 Faculty of Life Sciences, University of Manchester, Oxford Road, Manchester, M13 9PT, UK; 2 Bioinformatics & Evolutionary Genomics, VIB/Ghent University, Technologiepark 927, B-9052 Ghent, Belgium

## Abstract

**Background:**

Alternative representations of biochemical networks emphasise different aspects of the data and contribute to the understanding of complex biological systems. In this study we present a variety of automated methods for visualisation of a protein-protein interaction network, using the basic helix-loop-helix (bHLH) family of transcription factors as an example.

**Results:**

Network representations that arrange nodes (proteins) according to either continuous or discrete information are investigated, revealing the existence of protein sub-families and the retention of interactions following gene duplication events. Methods of network visualisation in conjunction with a phylogenetic tree are presented, highlighting the evolutionary relationships between proteins, and clarifying the context of network hubs and interaction clusters. Finally, an optimisation technique is used to create a three-dimensional layout of the phylogenetic tree upon which the protein-protein interactions may be projected.

**Conclusion:**

We show that by incorporating secondary genomic, functional or phylogenetic information into network visualisation, it is possible to move beyond simple layout algorithms based on network topology towards more biologically meaningful representations. These new visualisations can give structure to complex networks and will greatly help in interpreting their evolutionary origins and functional implications. Three open source software packages (InterView, TVi and OptiMage) implementing our methods are available.

## Background

"Graphics *reveal *data. Indeed graphics can be more precise and revealing than conventional statistical computations." Edward R. Tufte, *The Visual Display of Quantitative Information *[1].

The effective visual representation of complex data is an integral but perhaps undervalued part of a bioinformatician's job [2]. For an increasing number of researchers, this largely concerns the representation of networks, defined as sets of nodes (also called vertices) with corresponding sets of connections (undirected edges or directed arcs) between nodes. Methodologies that make the depiction of biological networks more accessible to biologists need to be developed in order to make these complex data sets as meaningful, and useful, as possible.

### Biological networks

Biological networks come in many shapes and sizes. Signalling networks, food webs, metabolic pathways and gene regulation networks are examples of network data sets that are models of biological systems, often encapsulating knowledge representing many decades of experimental work [3]. Other types of network are derived from computations on genomic data, via literature mining or from the results of high-throughput experiments, and are therefore only indirectly related to the underlying biological system [4]. This latter class would include gene co-expression and co-mention networks, most protein-protein interaction (PPI) data sets and networks constructed using phylogenetic profiles [5] or gene fusion data [6].

### Network layout algorithms

Network layout algorithms automatically produce visual representations of the linked nodes of a network. The aim of these algorithms is to provide easily interpretable layouts [7]. There are many aesthetic goals for these algorithms, including minimizing the number of edge crossings, minimizing the total area of the graph and maximizing symmetry [8]. Authoritative accounts of the wide variety of network layout algorithms are given in [9].

Both generic and bioinformatics-specific software are currently used for the visualisation of biological networks (Table 1). Such applications are often limited in the number of nodes and interactions that can be displayed clearly at the same time. They are capable of showing the topology of a network, but are usually devoid of meaningful biological context. Our aim here is to present some automated methods for visualisation of a protein-protein interaction network that incorporate biological information.

**Table 1:** Examples of network visualisation programs used in bioinformatics

**Software**	**Description**
Pajek [37]	A visualisation and analysis application for large graphs, used primarily for social network analysis.
GraphViz [38]	Implements a number of common graph layout algorithms.
Otter [39]	A tool developed for visualisation of internet data.
H3Viewer [40]	Provides layout and interactive navigation of graphs in three-dimensional hyperbolic space.
Biolayout(Java) [41]	Higher-level biological networks (e.g. metabolic pathways and regulatory networks) may be visualised. Basic network statistics are reported.
Osprey [42]	Builds data-rich graphical representations that are colour-coded for gene function and experimental interaction data. Web interfaces are used to retrieve up-to-date interaction data.
Cytoscape [43]	Integrates PPI networks with microarray and other gene expression data. Allows analysis of such networks by filtering subsets of nodes or interactions.
MAGE [44]	Used primarily in molecular modelling but has also been used to visualise networks. Produces three-dimensional images that can be zoomed and rotated in real time.
VisANT [45]	Developed to provide interactive visual mining of biological interaction data sets.
Java applet [46]	Displays protein interactions organised by network distance and biological function.

### Integration of biological information into network visualisation

The application of visualisation technology to network data can provide important insights into a system's structure and function [2]. In particular, integrating protein-protein interaction networks with supplementary information about the biological relationships between proteins makes it possible to display the network in a more meaningful way [10]. This extra information could be in the form of a phylogenetic tree, genomic location, known functional relationships, cellular compartments etc.

### The bHLH gene family

In order to explore the alternative methods by which network data may be organised meaningfully, using biological information, a data set was needed that was rich in protein interactions and additional information, such as phylogeny. Our data set of choice was the bHLH transcription factor family that was previously studied by our group [11].

The basic helix-loop-helix (bHLH) proteins are a complex multi-gene family of transcription factors with a wide role in the developmental processes of an organism, including neurogenesis, myogenesis, and sex determination [12]. The characteristic bHLH domain is approximately 60 amino acids long and has a DNA binding region followed by two α-helices, separated by a variable-length loop. This HLH domain promotes dimerization, allowing the formation of homodimers (a complex of two identical protein molecules) or heterodimers (a complex of two different proteins) between different group members [11]. bHLH proteins are found in eukaryotic lineages but not in prokaryotes. It is assumed that the animal bHLH group expanded by gene duplications at the origin of animal multi-cellularity [12]. Based on previous studies, the mammalian bHLH proteins have been classified into five sub-families according to both phylogenetic relationships and PPI network topology [11,13,14].

In our previous analysis of the evolution of the bHLH transcription factor family [11], it became apparent that the visualisation of the protein interaction network topology alone provides limited biological insight. The integration of phylogenetic data resulted in the network becoming more ordered and biologically more meaningful. During this work, it became clear that an automated visualisation tool was needed. Here we explore several alternative types of evolutionary information – pairwise sequence diversity, discrete phylogenetic groupings, the inferred evolutionary tree itself – and discuss the biological insight that can be implied from the different types of representation.

## Results

### Spring-embedded layout

**Figure 1:**
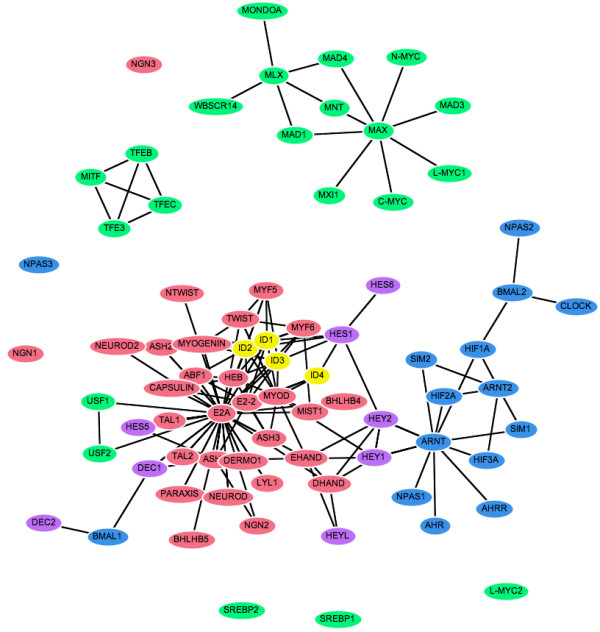
**Spring-embedded layout for the bHLH PPI network**. Nodes represent proteins and are coloured according to sub-family (or group): E2A/A group (red), MAX/B group (green), ARNT/C group (blue), ID/G group (yellow) and HES/E group (purple). Edges show reported physical interactions between proteins taken from [11].

Figure 1 shows a typical layout produced by our spring-embedded viewer, using only the network topology as input. Nodes are coloured according to their bHLH sub-family (or group). This representation of the network would be similar to the output of many of the tools for network visualisation listed in Table 1. Although this view emphasises the topological features of the PPI network, the nodes are not organised according to any extra biological information and in general the different colours are randomly scattered across the page.

### Spring-embedded layout incorporating evolutionary distances

**Figure 2:**
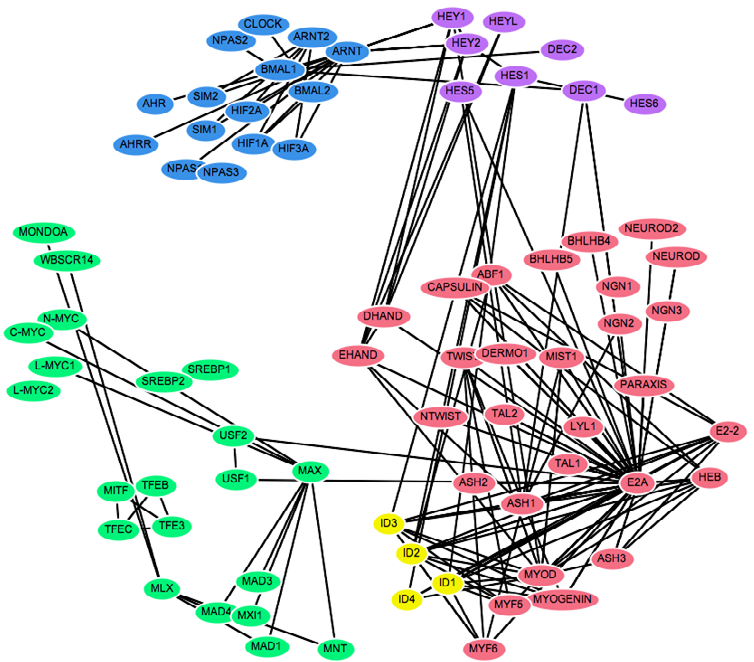
**Spring-embedded layout incorporating evolutionary distance information**. Nodes are automatically arranged so that the distance between proteins reflects their sequence divergence. The colours have the same designations as figure 1.

When we adapt our viewer to distribute nodes according to the evolutionary distances between protein sequences, the extra constraints make it more difficult for the spring-embedding algorithm to reach a globally optimal solution without manual assistance. However, repeated visualisations using random starting positions of the nodes gave qualitatively similar clustering of the bHLH sub-families within the PPI network, confirming that the method is reliable and reproducible. By dragging nodes around the screen it is possible to explore alternative network arrangements to determine if more stable layouts may be reached and to test how well such layouts agree with secondary information such as the sub-family groups. Figure 2 shows a typical output for the bHLH network. As expected the ARNT and HES groups (blue and purple) form distinct clusters, whilst E2A and ID (red and yellow) appear to be closely related, and the paraphyletic group MAX (shown in green) is more dispersed.

### Cluster-based layout

**Figure 3:**
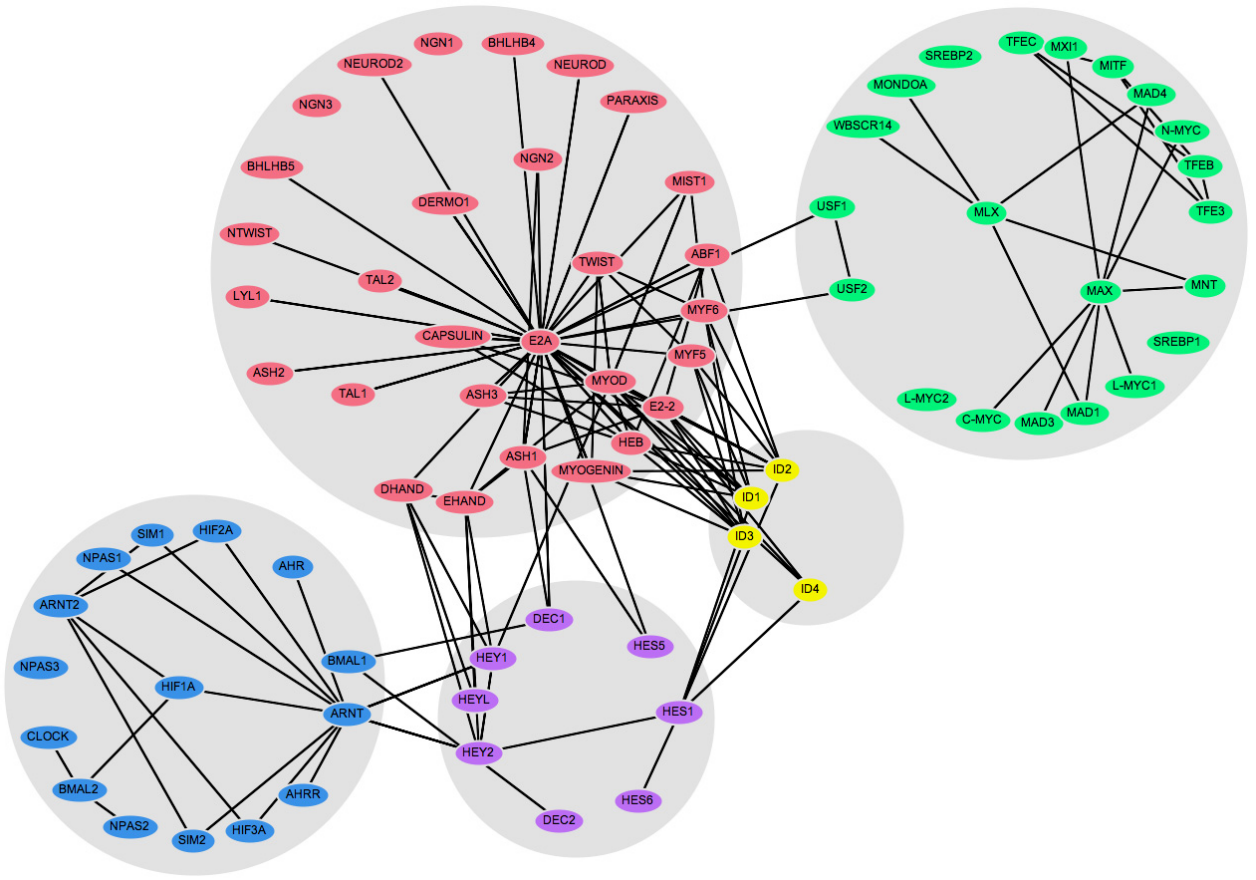
**Clustered layout of the bHLH interaction network**. Nodes are placed into discrete clusters or "containers" corresponding to sub-families. The colours have the same designations as figure 1.

In many circumstances, a known classification of the nodes in a network is available that can provide important biological context to the network visualisation. In these cases, it may be appropriate to partition the nodes into discrete clusters and present these as separate groups. We implemented such a view by a further adaptation of our spring-embedded network viewer, creating a "container" for each cluster within which nodes belonging to that cluster are constrained to lie. The network layout works in the same way as the original spring-embedded viewer, so that the nodes and their containers automatically arrange themselves on the screen to produce an easily interpreted view that can be manipulated by the user. Figure 3 shows the output of this layout program, where the bHLH PPI network has been clustered according to the identified protein groups. Each grey "container" circle has an area proportional to the number of nodes it contains.

### Phylogenetic interaction matrix

**Figure 4:**
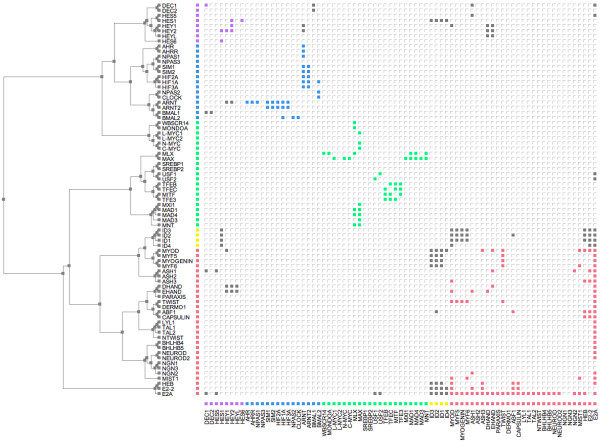
**Phylogenetic interaction matrix for the bHLH PPI network**. The inferred bHLH phylogenetic tree from [11] is shown on the left (branch lengths are not to scale). Intra-sub-family interactions are shown in the matrix to the right as filled cells in the same sub-family colour designations as figure 1; interactions between sub-families are in grey.

In contrast to the traditional nodes-and-edges view, a PPI network may also be represented as an interaction matrix, where the proteins are ordered in a list to reflect a phylogenetic tree and each cell represents a protein-protein interaction. This type of visualisation has been used in several recent publications [15-18]. The ATV tree viewer program [19] was modified to produce an interactive view of a phylogenetic matrix (Figure 4). Coloured cells represent interactions between proteins belonging to the same sub-family and grey cells the interactions between proteins in different sub-families. To help explore the relationship between the PPI data and the phylogeny, the tree may be rearranged by re-rooting at a selected node or by swapping the order of the branches.

### Tree and Arcs

**Figure 5:**
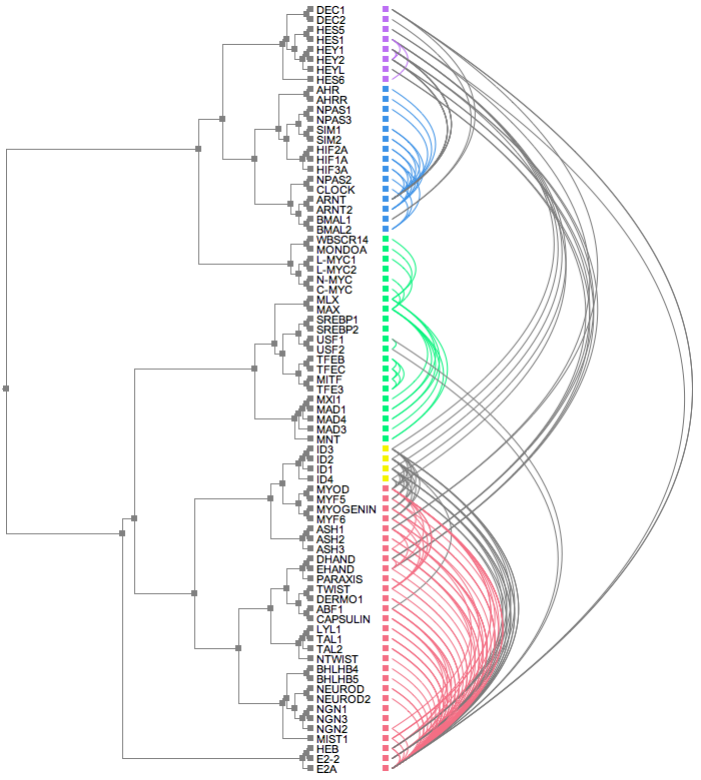
**Tree-and-arcs view of the bHLH PPI network**. The inferred bHLH phylogenetic tree from [11] is shown on the left. Intra-sub-family interactions are shown as arcs to the right and in the same sub-family colour designations as figure 1, interactions between sub-families are in grey.

To investigate another method for visualising protein interactions in relation to phylogeny, the ATV program [19] was further modified to display the protein-protein interactions as arcs against a phylogenetic tree. Figure 5 shows such a view of the bHLH PPI network. Grey arcs represent interactions between proteins in different sub-families; coloured arcs represent interactions within the same sub-family. The phylogenetic tree may be rearranged in the same way as for the matrix view, both to help explore the PPI network topology in relation to the tree and to minimise the number of arc crossings in the view.

### Tree layout in three-dimensions

**Figure 6:**
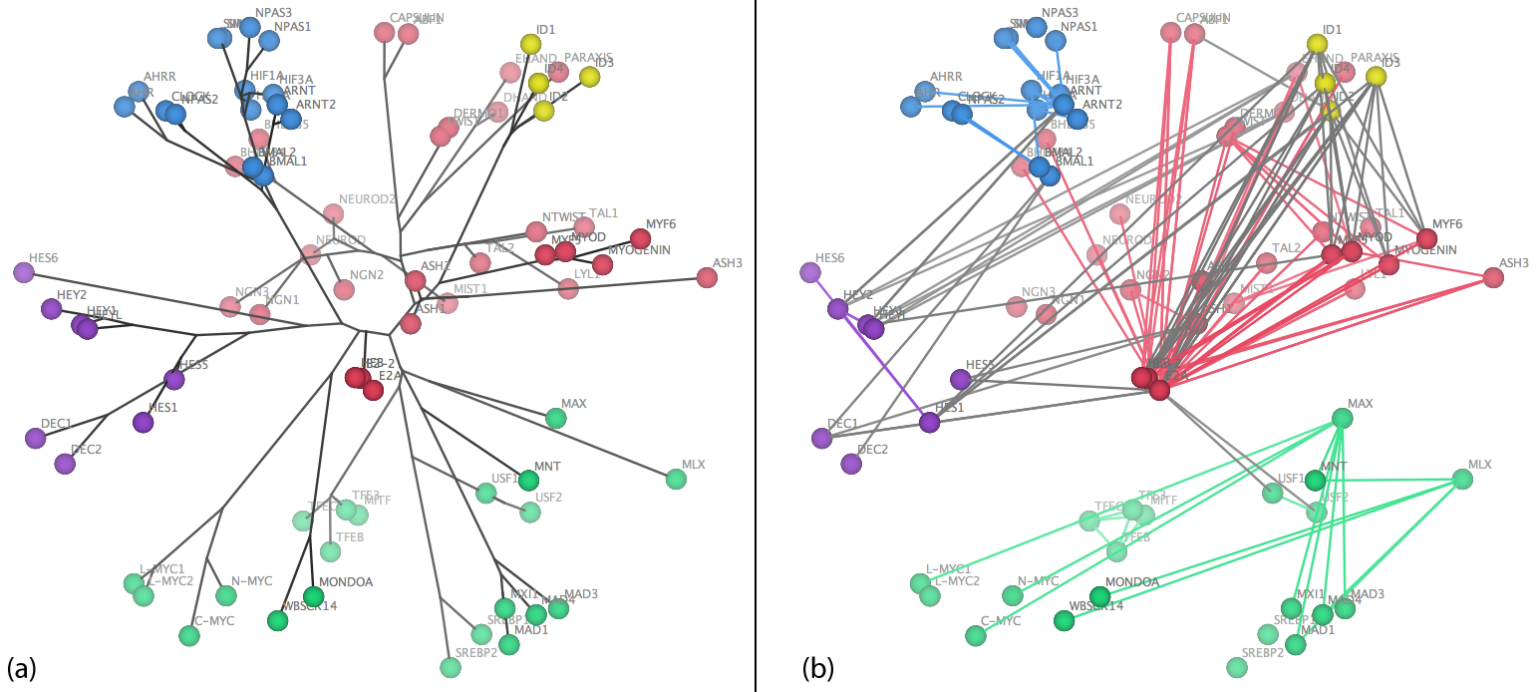
**Force-directed tree layout in three-dimensions**. Adding an extra dimension to the tree layout separates the sub-families according to evolutionary distance and makes their interactions easier to explore. The colours have the same designations as figure 1. For clarity the tree layout (A) and interactions (B) are shown separately. The three-dimensional Kinemage file is available as an additional file.

Using a force-directed optimisation method, we were able to produce tree layouts in three-dimensions upon which the protein interactions could be projected. This method is not guaranteed to find the globally optimal solution, so different random starting positions for nodes converge to different results. However, all runs produced final tree layouts that were qualitatively very similar, showing a clear separation of the bHLH sub-families in evolutionary space as shown in Figure 6. It is easy to explore the network structure in this view by rotating and zooming the layout (Additional File 1) using the KiNG three-dimensional visualisation software [20].

## Discussion

The methods used in this study have been chosen to illustrate how secondary information may be applied to organise a PPI network into a biologically meaningful visualisation. In principle, this extra information could be in the form of phylogenetic profiles, genomic location, functional similarity, cellular compartment, gene co-expression or any other discrete or continuous property of proteins (or pairs of proteins). Since in this case study we are primarily interested in using visualisation methods to investigate the evolution of interactions in the bHLH gene sub-family where proteins are paralogous, we have concentrated on the use of phylogenetic information, namely an evolutionary distance matrix and a phylogenetic tree.

### Evolutionary distances

Using evolutionary distance data as constraints between protein pairs within the PPI network (Figure 2) consistently produced a meaningful clustering of the proteins with respect to their sub-families. Our evolutionary distance visualisation of the bHLH network organises the nodes into well-defined groups that correspond to the protein sub-families. This can be taken as evidence that this method is successful in producing node arrangements that are meaningful representations of the evolutionary relationships between the proteins.

Ramani and Marcotte [17] used a similar method in three-dimensional space to organise PPI networks according to distance matrix data. These networks were used to substantiate that interacting protein partners exhibit correlated evolution and therefore have similar phylogenetic trees, since proteins that cluster in distinct regions in space mirror the adjacent placement of orthologues in a phylogenetic tree.

In the bHLH interaction network, the clusters formed by the different sub-families have been shown to have distinct functionalities [11], and it is in general true that a set of proteins organised according to sequence similarity can directly reveal protein function [10]. Protein interaction networks are already being used to uncover biological roles or functional classifications for unknown proteins, for example in the popular "guilt by association" method [21]. This gives rise to the notion of using network visualisations organised by evolutionary distances as exploratory tools for the prediction of function for unannotated proteins.

### Cluster-based layout

Grouping proteins according to secondary information provides an additional level of abstraction by which the PPI network may be organised (Figure 3). Partitioning the network in such a way clarifies the visualisation of interactions both within and between groups, and may help to resolve ambiguous group assignments. Discrete clustering may also be useful in cases where the biological property of interest is not subject to continuous variation, for example Gene Ontology functional category or DNA binding motif.

This view of the bHLH network emphasises the connectivity between the different groups, showing for example that the ARNT cluster (in blue) connects only with the HES group (purple), whereas the ID proteins (yellow) connect to both the HES and E2A (red) groups. The hubs within each group can also be clearly identified, and in general the layout is clearer, with fewer edge crossings and less clutter than the original spring-embedded layout (Figure 1).

Discretely partitioning the network allows us to apply many different types of secondary information to its layout. This becomes particularly important whenever biological networks are considered to have a modular structure [22]. By separating modules into different groups, the degree of modularity can be visualised objectively by the relative numbers of inter- and intra-module edges. Each module is clearly shown as a separate unit, operating in the context of the whole system.

### Phylogenetic trees

Drawing protein interactions against the phylogenetic tree maps function to phylogeny, permitting explicit interaction relationships within and between sub-families to be explored. This may also provide a visual insight into the evolutionary processes responsible for the PPI network [11]. Our automated implementation juxtaposes the interaction matrix against a phylogenetic tree (Figure 4). There are a number of features of the PPI matrix that make it an ideal tool for investigating network characteristics. For example, self-interactions – shown as filled cells on the diagonal of the matrix – are particularly clear using this representation. These interactions can be difficult to distinguish using other visualisation methods. Clusters of PPIs connecting proteins from the same sub-family are arranged near the diagonal and are coloured in the appropriate sub-family colour. Interactions that are between proteins in different sub-families are shown in grey. The relationship between the protein family phylogeny and the interaction matrix may also be explored by manipulating the tree layout. The tree may be re-rooted at a specified node, or branches swapped, thus preserving the tree topology whilst changing the order of the interaction matrix.

This phylogeny and matrix visualisation reveals hidden clusters of interactions, not apparent in other representations of the network. A further advantage of this method is that larger numbers of proteins and interactions can be mapped and visualised than in other representations, where high densities of interactions can obscure the detail of the network topology. Nodes with special characteristics can reveal themselves much more clearly in this representation than in a busy classical graph layout. Proteins acting as "hubs" are indicated by the presence of many filled cells within the same row or column, as seen for example with E2A and ARNT. A block of filled cells highlights interactions that have been retained by a group of closely-related proteins, for example the interactions of the "linker" proteins ID1 to ID4. Hongchao *et al*. [15] also successfully combined this technique with a clustering algorithm to visualise the PPI network in yeast. The interaction matrix representation of a PPI network is a good starting point for the exploration of very large graphs. Such visualisation is analogous to an aerial satellite map, providing a good overview of the complete network of interactions, which can then be "zoomed in" to explore the detail of specific areas.

In an alternative visualisation of the protein interactions in relation to phylogeny, the protein-protein interactions were drawn as arcs against the same phylogenetic tree (Figure 5). As in the matrix view, the interactions may be plotted against the entire tree or against a subtree in order to show more detail. An important first insight to the PPI network may be gained with this method, revealing the context of potentially important proteins such as hubs or linkers within the network. Although the tree-and-arcs view tends to be more cluttered than the matrix view, with arcs crossing and partially obscuring each other, it has a much greater visual impact and emphasises the number of connections to each individual node. This particular visualisation has been instrumental in revealing hidden order in the topology of another protein dimerisation network, that of the bZIP transcription factors: in particular it revealed a link between redox control of DNA binding and the architecture of the network [23]. Phylogenetic tree and network visualisation have also been combined in the TreeDyn package [24].

Moving to a three-dimensional representation of the phylogenetic tree (Figure 6) produces a clear separation of the proteins according to evolutionary distance. Although this view is probably the most information dense of all those considered in this study, use of an interactive viewer such as KiNG allows the network to be explored by zooming or rotating the view. In addition, the various components (tree, protein nodes, interactions and labels) may be displayed or hidden independently to emphasise different aspects of the data. Exploring other approaches to visualisation in three-dimensions [25,26] has the potential to be very useful for the layout of biological networks.

### Insights into the evolution of the bHLH transcription factors

Several models of evolution in PPI networks have been based upon ideas of gene duplication and domain reshuffling [27-30]. Other models have assumed that an existing ancestral network is duplicated when all genes coding for interacting proteins are duplicated simultaneously [31]. This may occur during whole-genome duplication, or tandem duplications, where a fraction of the interactions between duplications may become lost.

Our automated visualisations support an interesting mechanism of evolution of the bHLH network proposed by Amoutzias *et al*. [11], namely that the network has evolved its sub-family structure by single domain rearrangements and then duplication of these, rather than generation of new sub-families by large-scale duplication events such as whole genome duplication. Note, this does not preclude a role for large-scale gene duplication in increasing the total number of paralogues in the network, subsequent to the duplication of the precursor bHLH sub-family members. Each sub-family is characterised by a distinct domain arrangement, and most form well-defined phylogenetic groups. The exception to this is the MAX group (green), which is paraphyletic. The five sub-families of this network are distinguishable in all of the visualisations, though the MAX group is clearly more dispersed than the other clusters in the evolutionary distance representations (Figures 2 and 6) and forms two distinct clades in the phylogenetic tree (Figures 4 and 5). This evolutionary relationship indicates that the MAX (or B group) most probably corresponds to the ancestral group as defined by its shared domain architecture.

The alternative representations of the network also make the evolutionary origins of its topological structures much clearer than the basic spring-embedded view shown in Figure 1. For example, repeated duplication of the ancestral MAX-binding MYC and MAD proteins has led to MAX gaining a hub-like character. Figures 2 and 6 show the MYC and MAD proteins as distinct groupings, all attached to MAX. The same information is shown in Figure 4 by two localised groups of filled green cells in the MAX row, and in Figure 5 as two sets of parallel arcs all linking to MAX. Several other examples of the retention of protein interactions following gene duplication can be seen in these figures.

The cluster-based view emphasises the connectivity of the five sub-families (Figure 3). The HES group of repressors (purple) acts as a set of "bridge" proteins between the E2A (red) and ARNT (blue) sub-families. The ID repressors (yellow) mainly interact with the E2A group, but also bind HES1. Finally, the MAX group (green) is almost completely independent of the rest of the network: only USF1 and USF2 bind a protein from another sub-family (E2A).

## Conclusion

Although many different programs are available for the visualisation of networks in bioinformatics, a major disadvantage of these existing tools is their lack of biological context. Producing layouts based solely on network topology gives only the most basic impression of a network's functional implications, and may actually obscure important relationships between the biological entities represented.

In this study we have demonstrated that the application of alternative network visualisation techniques can reveal different aspects of what are usually very complex data sets. The incorporation of secondary information in the form of distance metrics, trees or discrete groupings of nodes can provide insights into evolutionary processes and may help to define modules within hierarchically-structured systems. Using complementary visualisations as exploratory tools will assist in the analysis of network data sets of all sizes and types, giving us the means to put bioinformatic networks into their proper biological perspective.

## Methods

### bHLH sequence and interaction data

The mammalian bHLH multiple sequence alignments and PPIs were taken from our earlier work [11]. The bHLH interaction data were collated from the published literature. Multiple sequence alignments were created using CLUSTAL W [32] and gap-stripped to remove columns consisting of more than 50% gaps. For the purposes of investigating the use of phylogenetic data as constraints for network visualisation, pairwise evolutionary distances were calculated from the multiple alignment using the Jones-Taylor-Thornton substitution model for amino acid replacements per site [33]. The resulting distance matrix was used to infer a phylogenetic tree with the program BIONJ [34].

### Spring-embedded layout

As an example of a classic spring-embedded network layout, a protein network viewer was developed based on the TouchGraph Java library [35]. TouchGraph attempts to optimise the network layout by minimising the lengths of edges whilst making nodes repel each other. Crucially, the user is able to manipulate the resulting layout by clicking and dragging nodes around the screen, following which the network will "relax" to its preferred shape. Network visualisations produced using TouchGraph may therefore be described as "semi-automated", in the sense that the user is able to assist the layout algorithm in producing an aesthetically pleasing result that remains consistent with the constraints imposed by spring embedding.

### Spring-embedded layout incorporating evolutionary distances

The evolutionary distance matrix calculated from the bHLH sequence alignment was used as the basis for a weighted all-against-all network where each edge is weighted by the evolutionary distance between the two proteins that it connects. This network was used as a secondary input to the TouchGraph-based layout program such that each edge in the all-against-all network had an equilibrium length proportional to its weight, but only the edges present in the original PPI network were made visible. The spring-embedded layout algorithm will therefore attempt to optimise the distance between each pair of proteins so that it reflects as closely as possible their estimated evolutionary distance.

### Cluster-based layout

A further method of visualisation was investigated for PPI networks that have been clustered into discrete groups using biological information. The protein viewer using the TouchGraph library was used as the basis of another spring-embedded network viewer that constrains nodes to lie within circular "containers", with area proportional to the number of nodes in the corresponding cluster. For the bHLH network being studied, proteins were clustered according to their sub-family as defined in [11].

### Phylogenetic interaction matrix

Another method of visualisation that provides a direct comparison between the PPI network and the phylogenetic data is to draw the protein-protein interactions against the phylogenetic tree itself. The ATV software for the layout and manipulation of trees [19] was extended to plot an interaction matrix of the bHLH PPI network, where proteins are listed in the order corresponding to a layout of the neighbor-joining phylogenetic tree. Each filled cell within the matrix represents an interaction between two proteins. Different colours are used to differentiate interactions within or between protein sub-families.

### Tree and Arcs

As an alternative to the phylogenetic matrix view, the ATV software was further adapted to allow protein-protein interactions to be directly visualised on a phylogenetic tree, represented as arcs going from source to target protein. As in the matrix view, arcs were coloured to differentiate inter- and intra-sub-family interactions.

### Tree layout in three-dimensions

The visualisation of evolutionary distances in the PPI network was extended to a third dimension by using the optimization procedure described below. This finds a distribution of nodes in three-dimensional space such that their pairwise inter-node distances are as close as possible to a set of defined constraints. The constraint between each pair of nodes was calculated as the sum of branch lengths between the corresponding nodes in the tree.

The optimization procedure for inter-node distances is based on a force-directed approach, but is designed to be inherently 'noisy', including a progressive damping factor so that the layout gradually converges towards an optimal configuration whilst avoiding spurious local minima.

For a network with *n *nodes and a set of (positive) pairwise constraints *c*_*ij*_, the method can be summarised as follows:

1. Start with a damping factor *f *= 0.5 and a random distribution of the nodes in three-dimensional space.

2. Repeat *n *times:

Set *t *= 0.

Choose a node *x *at random.

Calculate distances *d*_*xj *_from *x *to every other node.

Calculate the relative errors exj=|dxj−cxj|cxj
 MathType@MTEF@5@5@+=feaafiart1ev1aaatCvAUfKttLearuWrP9MDH5MBPbIqV92AaeXatLxBI9gBaebbnrfifHhDYfgasaacH8akY=wiFfYdH8Gipec8Eeeu0xXdbba9frFj0=OqFfea0dXdd9vqai=hGuQ8kuc9pgc9s8qqaq=dirpe0xb9q8qiLsFr0=vr0=vr0dc8meaabaqaciaacaGaaeqabaqabeGadaaakeaacqWGLbqzdaWgaaWcbaGaemiEaGNaemOAaOgabeaakiabg2da9maalaaabaWaaqWaaeaacqWGKbazdaWgaaWcbaGaemiEaGNaemOAaOgabeaakiabgkHiTiabdogaJnaaBaaaleaacqWG4baEcqWGQbGAaeqaaaGccaGLhWUaayjcSdaabaGaem4yam2aaSbaaSqaaiabdIha4jabdQgaQbqabaaaaaaa@4339@ compared to the target constraints.

For the node *y *with the greatest error *e*_*xy*_:

Add *e*_*xy*_^2 ^to *t*.

Calculate the vector v→
 MathType@MTEF@5@5@+=feaafiart1ev1aaatCvAUfKttLearuWrP9MDH5MBPbIqV92AaeXatLxBI9gBaebbnrfifHhDYfgasaacH8akY=wiFfYdH8Gipec8Eeeu0xXdbba9frFj0=OqFfea0dXdd9vqai=hGuQ8kuc9pgc9s8qqaq=dirpe0xb9q8qiLsFr0=vr0=vr0dc8meaabaqaciaacaGaaeqabaqabeGadaaakeaacuWG2bGDgaWcaaaa@2E33@_*xy *_that moves *x *directly towards (or away from) *y *to a point such that *d*_*xy *_= *c*_*xy*_.

Move *x *by the damped vector *f*v→
 MathType@MTEF@5@5@+=feaafiart1ev1aaatCvAUfKttLearuWrP9MDH5MBPbIqV92AaeXatLxBI9gBaebbnrfifHhDYfgasaacH8akY=wiFfYdH8Gipec8Eeeu0xXdbba9frFj0=OqFfea0dXdd9vqai=hGuQ8kuc9pgc9s8qqaq=dirpe0xb9q8qiLsFr0=vr0=vr0dc8meaabaqaciaacaGaaeqabaqabeGadaaakeaacuWG2bGDgaWcaaaa@2E33@_*xy*_.

3. Calculate q=tn
 MathType@MTEF@5@5@+=feaafiart1ev1aaatCvAUfKttLearuWrP9MDH5MBPbIqV92AaeXatLxBI9gBaebbnrfifHhDYfgasaacH8akY=wiFfYdH8Gipec8Eeeu0xXdbba9frFj0=OqFfea0dXdd9vqai=hGuQ8kuc9pgc9s8qqaq=dirpe0xb9q8qiLsFr0=vr0=vr0dc8meaabaqaciaacaGaaeqabaqabeGadaaakeaacqWGXbqCcqGH9aqpdaGcaaqaamaalaaabaGaemiDaqhabaGaemOBa4gaaaWcbeaaaaa@321E@, i.e. the root-mean-square value of *e*_*xy*_. This value serves as a proxy for the goodness of fit between the current configuration and the constraints.

4. If *q *is lower than any previously found value, store the current layout as the best.

5. If no improvement in *q *has been seen for 10 iterations, reduce *f *by 0.5%.

6. If no improvement in *q *has been seen for 100 iterations or the maximum number of iterations has been reached, report the best layout and stop.

7. Go to 2.

The resulting three-dimensional layout was output as a kinemage file [36] and visualised using the KiNG software [20].

## Availability and Requirements

Project name: Network Visualisation.

Project home page: . The methods described in this paper are implemented in the following open source software packages:

### InterView

This software (based on TouchGraph [35]) includes implementations of the two-dimensional spring-embedded, distance-constrained spring-embedded and cluster-based layouts. Nodes and/or edges may be coloured to represent different functions or other relevant information and both undirected and directed networks are supported. Layouts produced with InterView may be exported as PDF images. InterView may be downloaded from .

### TVi

This software (based on ATV [19]) includes implementations of the phylogenetic tree/matrix and tree/arcs layouts. Nodes may be coloured according to defined groupings and the tree layout may be manipulated by swapping adjacent branches. Layouts produced with TVi may be exported as PDF images. TVi may be downloaded from .

### OptiMage

This software produces visualisations of networks in three-dimensional space, allowing nodes to be distributed in one of three ways: according to network topology, using a phylogenetic tree (with or without associated branch lengths), or using distance matrix data. Directed or undirected interaction networks may be used, and subsets of nodes and/or edges may be coloured according to defined groupings. Output is presented in kinemage format, making it easy to share via the Web. OptiMage is available both as a command-line program and via a web server at .

Operating systems: Platform independent; Programming language: Java; Licence: GNU GPL (OptiMage), Apache-style open source (InterView/TVi).

## Authors' contributions

BJH and JWP wrote the interaction matrix and tree-and-arcs visualisation software (TVi). JWP wrote the TouchGraph spring-embedded viewer (InterView, based on a prototype written by BJH) and 3D optimization software (OptiMage, based on a prototype written by SCL). GDA provided the data for the bHLH family of proteins. DLR conceived and supervised the project with the help of SCL and GDA. JWP and BJH drafted the manuscript. All authors read and approved the final manuscript.

## Supplementary Material

Additional file 1 bHLH.kin. Kinemage file containing the interactive three-dimensional view corresponding to Figure 6. This file may be viewed using the freely available KiNG software [20]. Click here for file
